# A case report of fulminant eosinophilic myo-pericarditis related to checkpoint inhibitors complicated by acute heart failure and cardiac tamponade

**DOI:** 10.1093/ehjcr/ytaf400

**Published:** 2025-08-16

**Authors:** Yaqub Betz, Selveras Zayed, Francesco Moroni, Robin LeGallo, Antonio Abbate

**Affiliations:** Division of Cardiovascular Medicine, University of Virginia, PO Box 8000662, Charlottesville, VA 22908, USA; Division of Pathology, University of Virginia, 1215 Lee St., Charlottesville, VA 22908, USA; Division of Cardiovascular Medicine, University of Virginia, PO Box 8000662, Charlottesville, VA 22908, USA; Division of Pathology, University of Virginia, 1215 Lee St., Charlottesville, VA 22908, USA; Division of Cardiovascular Medicine, University of Virginia, PO Box 8000662, Charlottesville, VA 22908, USA

**Keywords:** Checkpoint inhibitors, Eosinophilic myo-pericarditis, Fulminant myocarditis, Case report

## Abstract

**Background:**

Immune checkpoint inhibitors (ICIs) have revolutionized cancer treatment but are associated with various adverse effects, including myocarditis, with mortality rates up to 50%. We report a case of fatal ICI-related fulminant eosinophilic myo-pericarditis complicated by tamponade in a 69-year-old man with metastatic lung adenocarcinoma.

**Case summary:**

Two weeks after receiving one dose of pembrolizumab, the patient presented with sudden chest pain and dyspnoea. Examination revealed tachycardia, hypotension, and hypoxia. Electrocardiogram showed a new right bundle branch block and ST depressions in the precordial leads with an elevated troponin I of 49.69 ng/mL. Echocardiogram demonstrated globally reduced function and pericardial effusion, suggesting impending tamponade. Despite aggressive resuscitative efforts, the patient rapidly decompensated and ultimately went into cardiac arrest and passed away. Autopsy was performed with pathology demonstrating necrotizing eosinophilic myocarditis related to ICIs. Other potential causes of eosinophilic myocarditis, such as drug hypersensitivity, were felt less likely given lack of drugs associated with eosinophilic myocarditis. Eosinophilic granulomatosis with polyangiitis and hypereosinophilic syndrome were also less likely based on American College of Rheumatology criteria as well as the absence of peripheral eosinophilia.

**Discussion:**

This case emphasizes the need for awareness of eosinophilic myo-pericarditis as a potential complication of ICI therapy. It underscores the value of early endomyocardial biopsy in unstable patients with suspected acute myocarditis, ‘fast-tracking’ treatment initiation. It also highlights the rapid progression of cardiac complications in ICI-related myocarditis and the potential for tamponade, emphasizing the low threshold for consideration of myocarditis and treatment in patients initiating or receiving ICIs.

Learning points

- Recognize the association between immune checkpoint inhibitor therapy and the development of myocarditis
- Consider early treatment with corticosteroids in patients with fulminant myocarditis associated with immune check point inhibitors

## Introduction

Immune checkpoint inhibitors (ICIs) have revolutionized cancer treatment but are associated with various adverse effects, including myocarditis, with mortality rates up to 50%. We present a case of fulminant eosinophilic myo-pericarditis with tamponade in a patient with metastatic lung adenocarcinoma with recent ICI use.

## Summary figure

**Figure:**
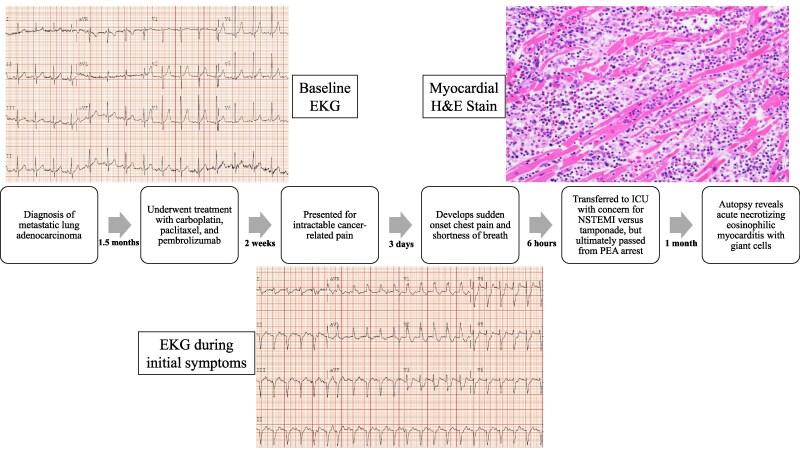


## Case presentation

A 69-year-old man with a history of ischaemic stroke 10 years prior complicated by post-stroke epilepsy treated with levetiracetam, active tobacco use with a 51-pack year smoking history, and metastatic lung adenocarcinoma was admitted for intractable cancer-related pain. With regard to his oncologic history, he was diagnosed 2 months prior to this admission. He had undergone treatment with one cycle of carboplatin, paclitaxel, and pembrolizumab 2 weeks prior to presentation.

He underwent intrathecal pain pump placement and received three rounds of radiation treatment to the pelvis and right scapula for pain relief without issue. Following a 3-day hospital admission, he was given discharge orders, and while waiting in his room, he suddenly developed chest pain and dyspnoea. Vitals were notable for new tachycardia with a heart rate of 123–137 b.p.m., hypotension with a blood pressure of 95/67 mmHg, and new hypoxia requiring a non-rebreather mask. His physical exam, which previously was largely unremarkable, was now notable for a patient in acute distress with significant tachycardia, jugular venous distention, tachypnoea, and cool, diaphoretic extremities. Assessment for pulsus paradoxus was negative.

**Figure 1:**
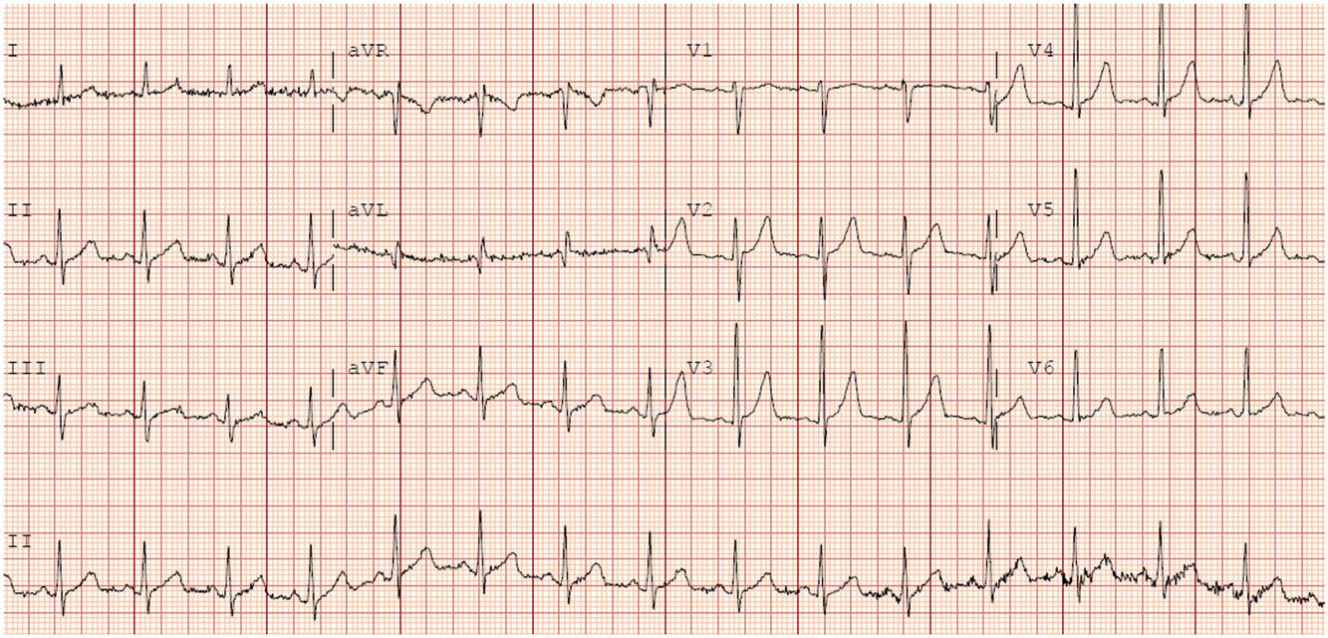
Electrocardiogram prior to admission.

**Figure 2:**
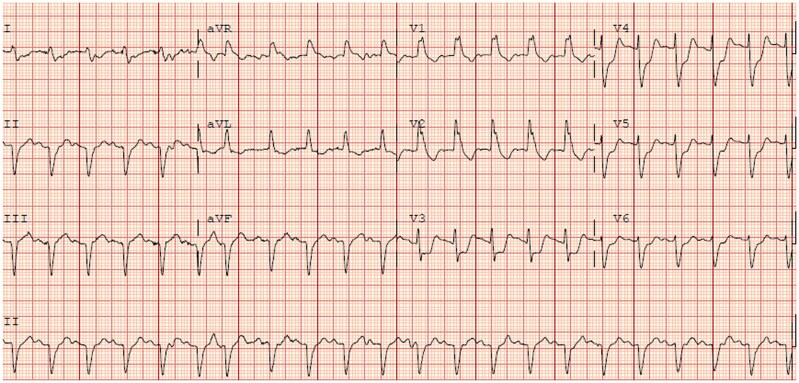
Electrocardiogram at time of initial decompensation.

Electrocardiogram demonstrated sinus tachycardia and new right bundle branch block as well as inferior pathologic Q waves and significant ST depressions in leads V_3_–V_6_ which were new from 3 weeks prior (*Figures 1* and *2*). Laboratory results were notable for rising liver enzymes with elevated aspartate transferase 221 U/L (normal < 35 U/L) and alanine transaminase 207 U/L (normal < 55 U/L), as well as elevated troponin I of 33.29 ng/mL that increased to 49.69 ng/mL (normal ≤ 0.120 ng/mL). Arterial lactic acid was also elevated at 3.90 mmol/L (normal < 2.2 nmol/L). There was a stable normocytic anaemia with a haemoglobin of 7.7 g/dL (normal range: 12–6 g/dL) and thrombocytopenia with platelets of 117•10^3^/µL (normal range: 150–450•10^3^/µL) and a white blood cell count of 9.11•10^3^/µL (normal range: 4–11 •10^3^/µL) with a differential of 71.0% neutrophils, 20.0% lymphocytes, 3.3% monocytes, 5.6% eosinophils, and 0.1% basophils. A transthoracic echocardiogram was performed which showed reduction in global ejection fraction from previously normal at 60–65% to 45–50% with a new, small pericardial effusion overlying the right ventricle with diastolic collapse of the right atrium and right ventricle, inferior vena cava plethora, and variation in mitral inflow velocities consistent with significant ventricular interdependence and impending tamponade. No significant valvular abnormalities were noted. Computed tomography angiography was performed and demonstrated no pulmonary embolism or other acute findings.

Within the subsequent hour, the patient was started on high-volume intravenous fluid resuscitation given concern for cardiac tamponade as well as aspirin and therapeutic anticoagulation for suspected non-ST-elevation myocardial infarction with plan for coronary angiogram and evaluation for pericardiocentesis. Unfortunately, within the next few hours, the patient continued to rapidly decline with increasing vasopressor and oxygen requirement as well as worsening mental status, and approximately 6 h after his initial decompensation, he went into cardiac arrest with pulseless electrical activity noted on the monitor. Cardiopulmonary resuscitation was attempted, but after discussion with family, resuscitative efforts were stopped resulting in death.

**Figure 3:**
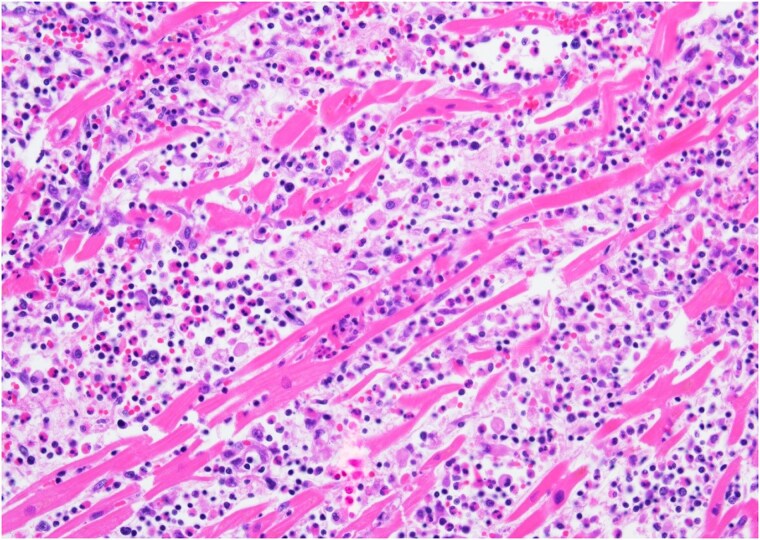
Mixed inflammation with prominent eosinophils, characterized by the binucleation and bright pink cytoplasm, with marked myocyte necrosis.

**Figure 4:**
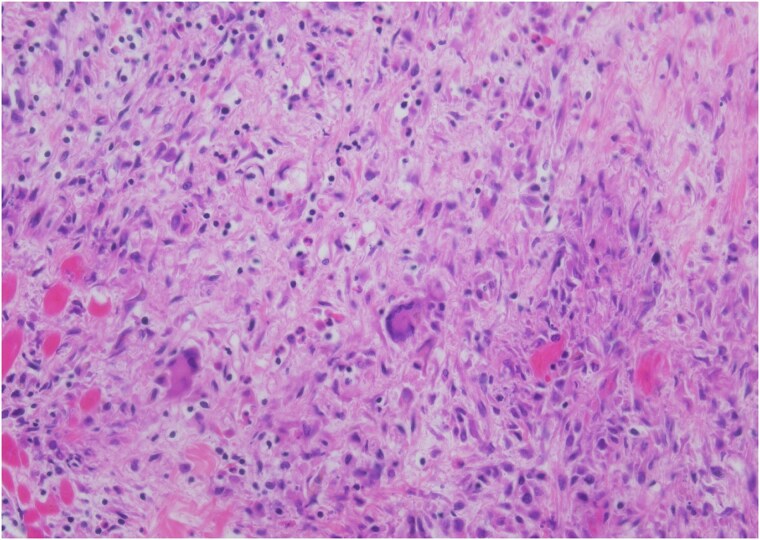
Advance lesion with granulation tissue and scattered multinucleated giant cells.

An autopsy was performed. Gross dissection demonstrated a structurally normal heart with four chamber dilation and mottling of the endomyocardium and papillary muscles. The coronary arteries showed moderate atherosclerosis with a maximum of 50% occlusion. Microscopic examination showed a diffuse interstitial inflammatory infiltrate comprised predominantly of lymphocytes and macrophages with numerous eosinophils with extensive oedema and myocyte necrosis involving all chambers (*Figure 3*). There were focal areas of organizing granulation tissue with scattered giant cells (*Figure 4*). The histologic features were most consistent with acute necrotizing eosinophilic myocarditis (EM) with giant cells.

## Discussion

Immune checkpoint inhibitors have revolutionized the treatment of malignancy but have also been shown to have a variety of adverse effects including acute myocarditis with mortality as high as 50% as well as pericarditis and pericardial effusion. Although the mechanism of ICI-related myocarditis is unknown, the current theories point to molecular mimicry that results in T-lymphocyte infiltration of the myocardium and can present on a spectrum from asymptomatic biomarker elevation to life-threatening fulminant myocarditis.^1^

In comparison, EM is a relatively rare form of myocarditis that, although is largely idiopathic, is associated with hypersensitivity syndrome from various drugs such as antibiotics, antiepileptics, and vaccines. The patient described herein had not been exposed to drugs associated with EM (*Table 1*). He had not had any recent vaccinations including to COVID-19 as he had declined vaccination. Although he was chronically treated with levetiracetam, it has not been associated with EM in the literature. When considering other causes of EM, both eosinophilic granulomatosis with polyangiitis (EGPA) and hypereosinophilic syndrome are possible. For the former, given the lack of peripheral eosinophils, history of asthma or obstructive airway, and neuropathy, EGPA was felt to be unlikely based on criteria from the American College of Rheumatology.^2^ Similarly, the lack of peripheral eosinophilia also made hypereosinophilic syndrome improbable. Therefore, as for the cause of this patient’s eosinophilic myo-pericarditis, checkpoint inhibitor therapy with pembrolizumab was considered the most likely diagnosis.

**Table 1:** Listing of pertinent prescribed medications

Acetaminophen
Amitriptyline
Aspirin
Dexamethasone
Enoxaparin
Ferrous sulfate
Gabapentin
Hydromorphone
Insulin lispro
Levetiracetam
Magnesium sulfate
Mirtazapine
Oxycodone
Polyethylene glycol
Senna
Sodium bicarbonate
Tamsulosin
Urea
Triamcinolone 0.1% ointment

This case reflects a rare example of ICI-related eosinophilic necrotizing myo-pericarditis with the presence of giant cells and represents an opportunity to expand our understanding of the potential cardiac toxicity of ICIs. Importantly, the presence of significant myocardial necrosis differentiates necrotizing EM from hypersensitivity EM and is associated with a more fulminant course as seen in this patient.^3^ Additionally, given the presence of giant cells, it appears to be a rare variant of EM typically associated with a severe degree of inflammation and poor clinical outcomes.^4^ Although tamponade is a rare complication of EM, it has been demonstrated in the literature^5,6^ that it is associated with ICIs and would potentially explain the pericardial effusion and rapid deterioration. The mortality of EM is high with rates of in-hospital death in one retrospective series of patients with EM at 22.3%. Although peripheral eosinophilia is commonly thought to be present in these patients, it may be absent in up to 25% of patients and is seen in high numbers in those with cardiac arrest and in-hospital death for unclear reasons.^7^

This case illustrates the diagnostic uncertainty of myocarditis and its potential for rapid decline. Guidelines recommend coronary angiography to rule out acute coronary syndrome, endomyocardial biopsy in high-risk patients (haemodynamically unstable, high-grade AV block, or sustained or symptomatic ventricular tachycardia, have heart failure refractory to treatment, or in whom drug toxicity is suspected), high dose IV corticosteroids, and consideration of mechanical circulatory support.^8^

In conclusion, we report a case of fatal ICI-related eosinophilic fulminant myo-pericarditis complicated by tamponade. The diagnosis of EM was made possible through pathology at time of autopsy. An awareness of the possibility of eosinophilic myo-pericarditis in patients with possible ICI-related myocarditis should prompt endomyocardial biopsy in high-risk subjects to facilitate the initiation of appropriate therapy.

## Lead author biography


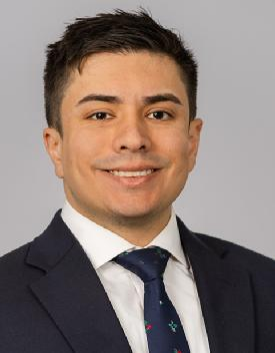


Yaqub Betz, MD, is a current third year cardiology fellow at the University of Virginia with a special interest in cardio-oncology and critical care. Pending completion of his fellowship, he will be joining the faculty at Thomas Jefferson Hospital in Philadelphia, Pennsylvania.

## Data Availability

The data underlying this article are available in the article and in its online supplementary material.

## Notes

**Consent:** The authors confirm that written consent for submission and publication of the clinical case including image(s) and associated text has been obtained from the patient in line with the COPE guidance.
